# Low cortisol levels in blood from dairy cows with ketosis: a field study

**DOI:** 10.1186/1751-0147-52-31

**Published:** 2010-05-20

**Authors:** Kristina B Forslund, Örjan A Ljungvall, Bernt V Jones

**Affiliations:** 1 Department of Clinical Sciences, Box 7054, Faculty of Veterinary Medicine and Animal Sciences, SLU, S-750 07 Uppsala, Sweden; 2 Kulterra AB, Rubens väg 7, S-740 21 Järlåsa, Sweden

## Abstract

**Background:**

An elevated plasma glucose concentration has been considered to be a potential risk factor in the pathogenesis of left-displaced abomasums (DA). Therefore the present study was performed to investigate if spontaneous disease (parturient paresis, metritis, ketosis etc) in dairy cows results in elevated concentrations of glucose and cortisol in blood as cortisol is the major regulator of glucose in ruminants.

**Methods:**

Cortisol, insulin, β-hydroxybutyric acid (BHBA), non esterified fatty acids (NEFA), and serum calcium were analyzed in blood serum and glucose, in whole blood, from 57 spontaneously diseased cows collected at different farms. The cows were grouped according to the disease; parturient paresis, recumbent for other reasons, mastitis, metritis, ketosis, inappetance and others.

**Results:**

No elevated concentrations of cortisol or glucose were found in cows with metritis and mastitis but both cortisol and glucose were elevated in cows stressed by recumbency. Cows with ketonemia (BHBA > 1.5 mmol/l) did not have low concentration of glucose in blood but significantly low levels of cortisol. Some of these cows even had cortisol concentrations below the detection limit of the analysing method (< 14 nmol/l).

**Conclusions:**

The study gives patho-physiological support to the treatment strategies of ketosis, recommending glucocorticoids, insulin etc. However further studies of this problem are needed to understand why cows with ketosis have low levels of cortisol and normal levels of glucose. To what extent elevated cortisol and glucose levels in hypocalcemic and recumbent cows are involved in the ethiology and /or the pathogenesis of DA also will need further research.

## Background

The reason for collecting field material to this study was a hypothesis that the known relation between stress and/or painful diseases in high yielding dairy cows and DA [1] may be mediated through a concurrent increased cortisol secretion leading to hyperglycaemia. Beside an increase in β-hydroxybuturate (BHBA), identified by LeBlanc et al. (2005) [2] as a possible metabolic predictor of DA in dairy cattle, an elevated plasma glucose level has been considered a potential risk factor for DA as hyperglycaemia has been shown to reduce the rate of abomasal outflow in dairy cows [3]. For many different reasons it was not possible to collect enough material to prove this hypothesis but when analysing the blood samples some of the results were interesting enough to be published.

In ruminants blood glucose regulation differs from the regulation in most mono-gastric animals. For example in humans, insulin is secreted after consumption of glucose but in ruminants there is a release of glucagon after fodder intake [4]. One regulator of glucose in ruminants is cortisol, which acts to increase gluconeogenesis from amino acids. In starving ruminants the gluconeogenesis is maintained by elevated levels of glucocorticoids [4].

In lactating ruminants the rate of hepatic gluconeogenesis and the relative concentrations of glycogenic precursors regulate the level of milk production [5,6]. The flow of glucose into the mammary gland is not dependent of insulin in ruminants [7,8].

Cortisol is used as an indicator of stress and pain and elevated serum cortisol has been shown in calves castrated without local anaesthesia [9], in surgical stress in dairy cows [10], in endotoxin mastitis and metritis [11,12], in cows suffering from inflammatory foot lesions [13] and in cows with hypocalcemia [14].

It can be hypothesized that the known relation between stressful periparturient diseases in high yielding dairy cows and DA [1] and the hyperglycaemia reducing the rate of outflow of abomasal fluid in dairy cows [3] may be mediated through a concurrent increased cortisol secretion leading to hyperglycaemia. This has, to our knowledge, not been previously studied.

The present study was undertaken to investigate possible associations between stressful and/or painful diseases in dairy cows and blood concentrations of glucose and cortisol and some other metabolites.

## Methods

### Animals

This study included 57 cows with spontaneous disease, prospectively recruited from the county of Uppland, Sweden. They were diagnosed suffering from either parturient paresis, mastitis, metritis, ketosis, recumbency, and others e.g. tetany, prolapsed uterus, laminitis and/or inappetance. The cows were housed at different farms and were in different stages of lactation. Following the call from a farmer the practitioner (one of the authors, Ö. L.) visited the farm and blood samples from the diseased cows were collected. The clinical examination was performed with history-taking, an ocular registration of general appearance, behaviour, body condition, respiration etc. Auscultations of heart, lungs, abdomen were performed and some percussion when indicated. All cows were checked for body temperature and udder health. The blood samples were drawn from the mammary vein using a Vacutainer^® ^(Becton, Dickinson, Franklin, NJ, USA) after the clinical examination but before initiation of treatments. The same veterinarian examined and diagnosed all sick animals in this study, took the blood samples and analyzed glucose at the farm before appropriate treatment was started. The recumbent cows with a suspicion of hypocalcaemia were treated with a Ca-infusion (Calphon^® ^vet. Bayer health care. Göteborg, Sweden) and re-sampled after about 15 min.

The dairy cows were grouped according to clinical diagnosis and concentration of calcium and β-hydroxybuturate (BHBA) in blood serum according to Radostits *et al*, (2007) [15] as outlined in Table 1.

**Table 1:** Grouping of diseased cows by diagnosis and blood serum concentration of calcium (S-tCa) and β-hydroxybuturate (BHBA)

					***AVERAGE^1 ^(95% CONFIDENCE INTERVAL) CONCENTRATION***	
					
***Group***	***Number of cows***	***Clinical diagnosis***	***Lactation number, median (range)***	***Production Cycle^2^, median (range)***	***S-tCa mmol/l***	***BHBA mmol/l***
IK	7	***Inappetance and ketosis***, BHBA > 1.5 mmol/l	2 (1;6)	26 (22;50)	2.2 (2.0;2.4)	4.5 (2.9;6.9)
I	5	***Inappetance***, BHBA < 1.5 mmol/l	5 (2;6)	2 (0;200)	2.1 (1.4;2.7)	0.9 (0.6;1.5)
MA	7	***Mastitis***	4 (2;6)	60 (3;250)	2.3 (1.8;2.8)	0.6 (0.4;0.9)
ME	7	***Metritis***	4 (2;4)	4 (3;14)	2.1 (1.7;2.5)	1.4 (0.7;2.5)
PP	12	***Parturient paresis ***S-tCa < 1.25 mmol/l	5 (2;8)	1 (0;60)	0.9 (0.8;1.0)	0.7 (0.5;0.9)
R	11	***Recumbent cows***, S-tCa > 1.25 mmol/l, treated with Ca. including prolapsed uterus, diarrhoea	4 (1;8)	2 (-1;200)	1.6 (1.4;1.9)	0.5 (0.3;0.9)
O	8	***Others***, including laminitis, tetany, vagus indigestion, "cough", no diagnosis, lymphadenitis	2 (1;5)	55 (1;180)	2.2 (2.0;2.4)	0.8 (0.3;2.3)

^1^Geometric means for BHBA, i.e. back-transformed from a logarithmic scale

^2^Days post partum

The sampling procedure was approved by the Ethical Committee for Animal Experiments, Uppsala, Sweden, according to the Swedish Act of Animal Welfare, 87/88.

### Analytical methods

The blood sample was analyzed immediately at the farm for glucose (Hemo-Cue, B-Glucosephotometer serial number 9540102191, Helsingborg, Sweden). The following blood serum components were determined at the clinical pathology laboratory of the Department of clinical sciences; non esterified fatty acids (NEFA) by an enzymatic method (NEFA C, Waco chemicals, Neuss, Germany), β-hydroxybuturate (BHBA) also by an enzymatic method (β-Hydroxybuturate, LiquiColor, Stanbio laboratory, Boerne, TX, USA). Serum total calcium (S-tCa) was analyzed by colourimetry (Konelab 30i chemistry analyser (Konelab corp, Espoo, Finland)). Cortisol was determined by a solid-phase RIA (DPC, Los Angeles, CA, USA, presently SMSD.) according to the manufacturer's instruction. The method has earlier been evaluated for use in bovine samples [16] (detection limit 14 nmol/l, intra-assay CV 2.2-6.3%,). For the purpose of statistical analysis concentrations of cortisol below detection limit were assigned a value of 7 nmol/l. Insulin was determined by RIA (Linco Research, St Charles, MO, USA).

### Statistical methods

The variables BHBA, glucose, insulin, cortisol and insulin/glucose were transformed to a logarithmic scale with base e prior to statistical analyses to get a normal distribution of the residuals sufficiently for the purpose of the analysis. The effect of clinical diagnosis on blood and serum components was analysed with a general linear regression model, with group of clinical diagnosis as the only explanatory variable. The model was validated by visual inspection of the distribution of the residuals and of identified outliers. Paired t-tests were used to study the effect of treatment of the paretic syndromes (parturient paresis and "recumbent" cow) on the blood and serum components.

## Results and Discussion

Average values of blood and serum components in the different groups are given in Table 2. Twenty one of the diseased cows had very low concentrations of cortisol i.e. below the detection limit (< 14 nmol/l). Five of these cows were in group IK (Inappetence and Ketosis, BHBA ≥ 1.5 mmol/l (5/7)) and four in group I (Inappetence, BHBA < 1.5 mmol/l (4/5)). The cortisol concentrations were highest in the samples from cows suffering from parturient paresis (mean 78.0 nmol/l, group PP) and recumbent cows (mean 96.7 nmol/l, group R). Both these groups had significantly higher (p < 0.001) levels of cortisol than the rest of the cows. The low concentration of cortisol in group IK and I were significantly lower (p < 0.03) than the cortisol level in group MA.

**Table 2:** Overall means^1 ^(95% confidence interval) of blood and serum components in the different disease groups before treatment according to clinical diagnosis ^2^, as estimated from linear regression model

	Clinical diagnoses						
	
	IK	I	MA	ME	PP	R	O
Glucose, mmol/l	2.9^a ^ (2.1-4.0)	3.4^a ^ (2.0-5.8)	3.3^a ^ (2.7-3.9)	3.2^a ^ (2.7-3.8)	5.2^b ^ (4.5-6.0)	6.1^b ^ (4.2-8.8)	3.1^a ^ (2.5-3.9)
Insulin, mU/l	3.7^a ^ (1.8-7.4)	6.7^ab ^ (1.6-27.9)	12.9^b ^ (6.9-24.2)	4.9^a ^ (1.4-17.8)	4.6^a ^ (3.3-6.3)	8.6^ab ^ (3.8-19.8)	8.8^ab ^ (3.6-21.6)
Cortisol, nmol/l	9.6^a ^ (5.7-16.1)	8.8^a ^ (4.7-16.6)	23.3^b ^ (7.4-72.9)	12.2^ab ^ (4.4-34.3)	78.0^c ^ (58.8-103.4)	96.7^c ^ (66.1-141.4)	15.8^ab ^ (6.9-36.3)
NEFA^3^, mmol/l	0.8^a ^ (0.1-1.6)	0.5^ab ^ (0.1-1.0)	0.4^b ^ (0.2-0.7)	0.5^ab ^ (0.3-0.8)	0.8^a ^ (0.6-1.0)	0.7^ab ^ (0.4-1.0)	0.4^bc ^ (0.2-0.6)

^1^Geometric means for glucose, insulin and cortisol, i.e. back-transformed from a logarithmic scale

^2^See Table 1 for definition of groups of clinical diagnoses

^3^NEFA = Non-esterified fatty acids

Numbers within row with a common letter in the superscript does not differ significantly (p > 0.05).

For the first time to our knowledge, low to not even measurable concentrations of cortisol have been shown in cows suffering from spontaneously occurring ketosis (BHBA > 1.5 mmol/l) [15], but this was not accompanied by low concentrations of blood glucose (> 2.9 mmol/l). Cows suffering from metritis, mastitis or other diseases causing pain had no elevated concentrations of cortisol or glucose in the blood. However, both cortisol and glucose were elevated in cows stressed by recumbency.

The accuracy in studies based on clinical cases may have a problem in the different clinicians' ability to specifically diagnose a disease. In this study it is a great advantage that the same clinician has determined the diagnoses, which to the main part later were verified by laboratory analyses.

Although group IK had ketosis (> 1.5 mmol of BHBA) and a mean NEFA level of 0.8 mmol/l they were not hypoglycaemic, neither was group I. Both these groups had low concentrations of cortisol, which was not expected since "starving" ruminants should maintain the gluconeogenesis with elevated levels of glucocorticoids [4]. Also Moyes *et al*, [17] presented higher serum cortisol concentration in negative energy balanced cows compared to cows with an *ad libitum *diet. Peripheral cortisol concentrations exhibit weak circadian rhythms in lactating cows with periods around 120 min [18], still, in spite of the low number of cows in each group; there were significant differences in cortisol levels between the groups in this study. Cortisol is synthesized from cholesterol in the adrenal glands which can import cholesterol from low-density lipoproteins (LDL)-cholesterol by means of LDL receptor mediated endocystosis or it can be synthesised *de novo *from acetate [19]. The milk production is driven by fodder such as for instance grain that produces glucose precursors in the rumen. Thus a lot of propionate and lactate is produced in rumen maybe on expense of acetate. Beerda et al [20] have shown that high yielding dairy cows have lower cortisol response to ACTH administration than low yielding cows. Lactation ketosis results from more factors than solely severe energy deficit [21]. One possible reason could be difficulties in synthesising cortisol when their energy demands increases in peak lactation.

Cortisol concentrations are said to peak sharply around parturition and decrease to the same level as prepartum concentrations after 3-5 days of lactation [22,23] or increase in dairy cows a few days before parturition and then have a significant decrease around 10 days post parturition [22].

The serum cortisol levels were highest in serum from recumbent cows (96.7 nmol/l) and cows suffering from parturient paresis (78.0 nmol/l) in the present study. Both these groups had significantly higher (p < 0.001) levels of cortisol than the rest of the cows and most of the cows in both these groups were close to parturition. Glucocorticoids also interfere with the absorption of Ca from the gastrointestinal tract [19]. The hypothesis that cows suffering from stress and/or painful diseases have elevated blood glucose levels due to an increase in serum cortisol could not be verified, as only cows suffering from paralysis (hypocalcaemia, group PP, and recumbent cows, group R) showed high blood glucose and serum cortisol concentrations compared to the others. The cows with hypocalcaemia (group PP) also had low concentrations of insulin despite the fact their levels of glucose were significantly higher than normal, indicating that they were unable to secrete insulin. This may be a result of the hypocalcaemia as insulin is secreted by a Ca-dependent exocytosis. After treatment with calcium solution the insulin levels were significantly (p < 0.02) increased (Table 3). Cortisol has an indirect antilipolytic effect in vivo but do not aggravate or accelerate fatty degeneration of the liver [24]. Still, one of the most frequent necropsy findings in cows around parturition is fatty liver [15].

**Table 3:** Overall means^1 ^(95% confidence interval) of blood components before and after treatment of cows with parturient paresis and of "recumbent" cows^2^

	Parturient paresis		"Recumbent" cow	
		
	Before	After	Before	After
Glucose, mmol/l	5.2^a ^ (4.5;6.0)	5.8^a ^ (5.1;6.6)	6.1^a ^ (4.2;8.8)	6.7^a ^ (4.8;9.5)
Insulin, mU/l	4.6^a ^ (3.3;6.3)	8.0^b ^ (4.6;14.2)	8.6^a ^ (3.8;19.8)	8.7^a ^ (4.0;18.9)
Cortisol, nmol/l	78.0^a ^ (58.8;103.4)	64.2^b ^ (53.1;77.6)	96.7^a ^ (66.1;141.4)	91.5^a ^ (63.0;133.1)
NEFA^3^, mmol/l	0.8^a ^ (0.6;1.0)	0.8^a ^ (0.6;1.0)	0.7^a ^ (0.4;1.0)	0.7^a ^ (0.5;0.9)

^1^Geometric means for glucose, insulin and cortisol, i.e. back-transformed from a logarithmic scale

^2^See Table 1 for definition of the diagnoses

^3^NEFA = Non-esterified fatty acids

Numbers within row and pair of columns with a common letter in the superscript does not differ significantly (p > 0.05)

The cows with metritis and mastitis did not have higher blood concentrations of glucose than the other groups (Table 2). Nor did cows suffering from ketosis have decreased concentration of glucose. Reference values often differ between studies, due to different methods of analysis being used, but it is generally agreed that the blood glucose concentrations in dairy cows are higher before parturition than after [25]. However, in the present study cows with mastitis were in different stages of lactation and had significantly higher levels of insulin than the rest of the cows indicating that the pancreas in these animals was stimulated by more glucose eventually due to decreased glucose drainage to the udder.

Cows stressed by recumbency (groups PP and R) had significantly higher levels of cortisol and glucose. Elevated plasma cortisol during spontaneous hypocalcaemia in ruminants has also been shown previously by Horst and Jörgensen [26]. A relationship between milk fever and DA in cows has been described by van Dorp *et al *[27], and experimentally induced hyperglycaemia is reported to significantly reduce the rate of outflow of abomasal fluid in dairy cows [3]. The plasma glucose levels used in that experiment were similar to the ones observed in the PP and R in our study. If the hyperglycaemia at parturition seen in cows with parturient paresis is high enough to influence the abomasal emptying rate later in early lactation is hard to say. We have no further information about DA from the cows in our PP or R groups.

The BHBA concentration in blood (excluding group IK with BHBA > 1.5 mmol/l) was significantly higher (p < 0.03) in group ME (with an average of 1.4 mmol/l) compared to group MA, (average 0.6 mmol/l) and group PP. LeBlanc *et al *[2] identified metabolic predictors of DA in dairy cattle and found that an increased BHBA concentration was the best predictor compared to NEFA, glucose, urea, Ca and P_in_. Metritis seems often to be a painful condition in cows with clinical signs as groaning, moaning and kicking the abdomen. In the present study we could not confirm any elevated concentrations of cortisol due to pain, but our study support, to a certain extent, LeBlanc *et al *[2] in their assumption that the BHBA might be a predictor of DA as metritis is considered to be one of several risk factors for DA [1].

The findings in this study of low cortisol concentrations in cows with clinical ketosis give a pathophysiological support to the treatment strategies of ketosis in veterinary medicine textbooks recommending glucocorticoids. However further studies of this problem are needed to understand why cows with clinical ketosis do not eat and have low levels of cortisol.

## Conclusions

No elevated concentrations of cortisol or glucose were found in cows with metritis and mastitis but both cortisol and glucose were elevated in cows stressed by recumbency. Surprisingly cows with ketosis (BHBA > 1.5 mmol/l) did not have low concentration of glucose in blood but significantly low levels of cortisol. Some of these cows even had cortisol concentrations below the detection limit of the analysing method (< 14 nmol/l).

The study gives patho-physiological support to the treatment strategies of ketosis, recommending glucocorticoids. However further studies of this problem are needed to understand why cows with ketosis have low levels of cortisol and normal levels of glucose. To what extent elevated cortisol and glucose levels in hypocalcemic and recumbent cows are involved in the ethiology and/or the pathogenesis of DA also will need further research.

## Competing interests

The authors declare that they have no competing interests.

## Authors' contributions

KF initiated the study, contributed with the expertise in metabolic diseases in ruminants and was responsible for the manuscript preparation. ÖL collected the blood samples after diagnosing the cows and then analysed blood glucose. BJ was responsible for analyzing the rest of the blood serum components and contributed with expertise concerning those. All authors read and approved to the final manuscript.

## References

[B1] Rohrbach BW Cannedy AL Freeman K Slenning BD Risk factors for abomasal displacement in dairy cows J Am Vet Med Assoc 1999 214 1660 3 10363100

[B2] LeBlanc SJ Leslie KE Duffield TF Metabolic predictors of displaced abomasum in dairy cattle J Dairy Sci 2005 88 159 170 10.3168/jds.S0022-0302(05)72674-6 15591379

[B3] Holtenius K Sternbauer K Holtenius P The effect of plasma glucose level on abomasal function in dairy cows J Anim Sci 2000 78 1930 1935 10907836 10.2527/2000.7871930x

[B4] Trenkle A Endocrine regulation of energy metabolism in ruminants Fed Proc 1981 40 2536 41 7021187

[B5] Lomax MA Baird GD Blood flow and nutrient exchange across the liver and gut of the dairy cow. Effects of lactation and fasting Br J Nutr 1983 49 481 496 10.1079/BJN19830057 6860627

[B6] Huntington GB Energy metabolism in the digestive tract and liver of cattle: influence of physiological state and nutrition Reprod Nutr Dev 1990 30 35 47 10.1051/rnd:19900103 2184823

[B7] Zhao F-Q Keating AF Expression and regulation of glucose transporters in the bovine mammary gland J Dairy Sci 2007 90 E Suppl E76 E86 10.3168/jds.2006-470 17517754

[B8] Nielsen MO Madsen TG Hedeboe AM Regulation of mammary glucose uptake in goats: role of mammary gland supply, insulin, IGF-1 and synthetic capacity J Dairy Res 2001 68 337 349 10.1017/S002202990100499X 11694037

[B9] Thüer S Mellema S Doherr MG Wechsler B Nuss K Steiner A Effect of local anaesthesia on short- and long-term pain induced by two bloodless castration methods in calves Vet J 2007 173 333 42 10.1016/j.tvjl.2005.08.031 16223591

[B10] Mudron P Scholz H Sallmann HP Rehage J Kovac G Bartko F Holtershinken M Effect of vitamin E injection on cortisol and white blood cell response to surgical stress in dairy cows Int J Vitam Nutr Res 1994 64 176 180 7814231

[B11] Huszenicza G Janosi S Gaspardy A Kulcsár Endocrine aspects in pathogenesis of mastitis in postpartum dairy cows Anim Reprod Sci 2004 83 389 400 Review. 10.1016/j.anireprosci.2004.04.029 15271468

[B12] Kulcsar M Janosi S Lehtolainen T Kátai L Delavaud C Balogh O Chilliard Y Pyörälä S Rudas P Huszenicza Gy Feeding-unrelated factors influencing the plasma leptin level in ruminants Domest Anim Endocrinol 2005 29 214 226 Review. 10.1016/j.domaniend.2005.03.008 15885961

[B13] Almeida PE Weber PS Burton JL Zanella AJ Depressed DHEA and increased sickness response behaviors in lame dairy cows with inflammatory foot lesions Domest Anim Endocrinol 2008 34 89 99 10.1016/j.domaniend.2006.11.006 17229542

[B14] Waage S Sjaastad OV Blom AK Plasma concentrations of cortisol in cows with hypocalcaemia in relation to their responses to treatment with calcium Res Vet Sci 1984 36 164 8 6718815

[B15] Radostits OM Gay CG Hinchcliff KW Constable PD eds Chapter 29, Metabolic Diseases Veterinary Medicine 2007 10 Saunders Elsevier 1613 1690

[B16] Bolaños JM Molina JR Forsberg M Effect of blood sampling and administration of ACTH on cortisol and progesterone levels in ovariectomized zebu cows (Bos indicus) Acta Vet Scand 1997 38 1 7 9129341 10.1186/BF03548502 PMC8057030

[B17] Moyes KM Drackley JK Salak-Johnson JL Morin DE Hope JC Loor JJ Dietary-induced negative energy balance has minimal effects on innate immunity during a *Streptococcus uberis *mastitis challenge in dairy cows during midlactation J Dairy Sci 2009 92 4301 4316 10.3168/jds.2009-2170 19700690

[B18] Lefcourt AM Bitman J Kahl DS Wood DL Circadian and ultradian rhythms of peripheral cortisol concentrations in lactating dairy cows J Dairy Sci 1993 76 2607 12 8227661 10.3168/jds.S0022-0302(93)77595-5

[B19] Barret J The adrenal gland (Chapter 49) Medical Physiology 2003 Saunders, Elsevier Science ISBN: 0-7216-3256-4.

[B20] Beerda B Kornalijnslijper JE Werf JT van der Noordhuizen-Stassen EN Hopster H Effects of milk production capacity and metabolic status on HPA function in early postpartum dairy cows J Dairy Sci 2004 87 2094 102 10.3168/jds.S0022-0302(04)70027-2 15328221

[B21] de Boer G Trenkle A Young JW Glucagon, Insulin, Growth Hormone, and Some Blood Metabolites During Energy Restriction Ketonemia of Lactating Cows J Dairy Sci 1985 68 326 37 10.3168/jds.S0022-0302(85)80829-8 3886731

[B22] Goff JP Kehrli ME Jr Horst RL Periparturient hypocalcemia in cows: prevention using intramuscular parathyroid hormone J Dairy Sci 1989 72 1182 1187 10.3168/jds.S0022-0302(89)79222-5 2745825

[B23] Patel OV Takahashi T Takenouchi N Hirako M Saski N Domeki I Peripheral cortisol levels throughout gestation in the cow: effect of stage of gestation and foetal number Br Vet J 1996 152 425 32 10.1016/S0007-1935(96)80036-4 8791850

[B24] Furll M Jackel F Effects of corticoids on parameters of lipid metabolism, hepatic metabolism, haematological parameters and milk yield in high-yielding cows in early lactation Berl Munch Tierarztl Wochenschr 2005 118 247 54 15918490

[B25] Holtenius K Agenäs S Delavaud C Chilliard Y Effects of feeding intensity during the dry period. 2. Metabolic and hormonal responses J Dairy Sci 2003 86 883 891 10.3168/jds.S0022-0302(03)73671-6 12703625

[B26] Horst RL Jorgensen NA Elevated plasma cortisol during induced and spontaneous hypocalcaemia in ruminants J Dairy Sci 1982 65 2332 2337 10.3168/jds.S0022-0302(82)82505-8 6819310

[B27] van Dorp RT Martin SW Shoukri MM Noordhuizen JP Dekkers CM An epidemiologic study of disease in 32 registered Holstein dairy herds in British Columbia Can J Vet Res 1999 63 185 92 10480460 PMC1189546

